# Effect of Light Quality on the seed Germination and Development of Coffee Seedlings (*Coffea arabica*)

**DOI:** 10.3390/plants13131772

**Published:** 2024-06-27

**Authors:** Ana Julia Cardoso-Magaña, Mayra Itzcalotzin Montero-Cortés, Joaquin Alejandro Qui-Zapata, Arturo Moises Chávez-Rodríguez, Norma Alejandra Mancilla-Margalli, Julio Cesar López-Velázquez, Vania Sbeyde Farias-Cervantes, Isaac Andrade-González, Diana Maria Rivera-Rodríguez, Fabiola Bejarano-Rebolledo

**Affiliations:** 1 Tecnológico Nacional de México/Instituto Tecnológico de Tlajomulco, Km. 10 Carretera Tlajomulco-San Miguel Cuyutlán, Tlajomulco de Zúñiga 45640, Mexico; cardosomaganaanajulia@gmail.com (A.J.C.-M.); arturo.cr@tlajomulco.tecnm.mx (A.M.C.-R.); norma.mm@tlajomulco.tecnm.mx (N.A.M.-M.); vania.fc@tlajomulco.tecnm.mx (V.S.F.-C.); isaac.ag@tlajomulco.tecnm.mx (I.A.-G.); diana.rr@tlajomulco.tecnm.mx (D.M.R.-R.); fabiola.br@tlajomulco.tecnm.mx (F.B.-R.); 2 Biotecnología Vegetal, Centro de Investigación y Asistencia en Tecnología y Diseño del Estado de Jalisco A.C., Camino Arenero 1227, El Bajío, Zapopan 45019, Mexico; jqui@ciatej.mx; 3 Department of Bioengineering, School of Engineering and Science, Tecnológico de Monterrey, Campus Guadalajara, Ramón Corona 2514, Nuevo México, Zapopan, Jalisco 45201, Mexico; juliolv@tec.mx

**Keywords:** in vitro, *Coffea arabica*, light quality, ZG medium, ZP medium, germination, development

## Abstract

Coffee (*Coffea arabica*) cultivation is vital to the global economic, social and cultural life of farmers. However, senescent and disease-susceptible plantations affect coffee productivity. Therefore, it is crucial to improve biotechnological strategies such as micropropagation to increase the number of plants for replanting. In this study, the dark condition (T1) and different light qualities (T0-white light 400–700 nm; T2-red light 660 nm and T3-blue light 460 nm) were evaluated to optimize the in vitro propagation of 4 and 9 month-old coffee seeds. The results showed that red light had the highest percentage, an outstanding germination rate index, which may suggest that in the case of coffee seeds could be involved phytochromes that promote germination in a red light quality. In summary, the ideal conditions for in vitro micropropagation of coffee are under white and red light condition.

## 1. Introduction

Coffee is of great importance worldwide because of its commercial value, with total world production in coffee year 2023/24 amounting to 10,285 million tonnes. The main producers of Arabica coffee are Brazil, Colombia, Ethiopia and Honduras, while México ranks tenth in production with 245 million tonnes [1]. Mexico has the ideal agroecosystems for coffee cultivation, mainly in the centre-south of the country, covering an area of 702,686.02 ha [2]. The main producing states are Chiapas, Veracruz, Puebla, Oaxaca and Guerrero [3]. Mexico has ideal condition for coffee cultivation, with mountainous areas in the southeast of the country at altitudes of over 900 m, as well as temperatures in the range of 17.5 to 25.3 °C. Coffee growing in the country plays a key role in the agricultural sector, not only because of the value of its production and its contribution as a foreign exchange earner, but also because of its significant environmental benefits. In particular, 99% of coffee farm are established under native shade trees, which improves growing condition, helping to reduce the adverse impacts of climate change on coffee trees [4,5,6].

As for the coffee genus, the most commercial species are *Coffea canephora* and *Coffea arabica*. In Mexico, 13% (32.7 billion tonnes) of production correspond to *Coffea canephora*, while 86.5% (212.7 billion tonnes) is *Coffea arabica* [1]. The latter has desirable physical attributes, such as larger bean size, greater colouring, a chemical composition that gives it an intense aroma, high acidity, light body, which differentiate it in terms of beans quality compared to robust genotypes [7,8]. However, *Coffee arabica* has a low genetic diversity [9,10], which is reflected in high susceptibility to pest and disease [11,12]. 

Since the introduction of coffee in the country, significant change has been experienced largely due to disease caused mainly by fungi, with rust (*Hemileia vastatrix*), ranked as one of the 10 most devasting disease worldwide, affecting coffee crops in México since 1981. This problem has become a priority, concentrating considerable human, physical and economic efforts to combat this disease. However, there are also other endemic diseases of regional importance that affect different stage of the crop and contribute to limiting the development and production of the plant [13]. Therefore, it is essential to develop micropropagation techniques that guarantee healthy plants, creating optimal conditions for their development and free of pathogens. This will not only increase the yield and number of plants, but also improve the control of crop health status.

Coffee plants have both asexual reproduction processes; asexual reproduction generates individuals genetically identical to the parent without the intervention of gametes, while sexual reproduction involves the formation of seed, which ensure the perpetuity of the species [14]. *Coffea arabica* is mainly propagated by self-pollination, maintaining 90% of the original characteristics of the variety [15]. For this method, it is crucial to collect ripe and healthy fruits, prioritising the central beans of coffee trees and branches, avoiding unripe, damage or pest-affected fruits [16]. Coffee seeds have been considered recalcitrant, orthodox and even intermediate with varying results and sensitive to drying [17]. One cause of the decrease in the germination percentage of coffee seeds is the decrease in water content due to drying after harvest. Drying can increase the levels of reactive oxygen species (ROS) as a result of physiological stress. Coffee seeds that have been stored will undoubtedly experience a period of seed dormancy; a method is needed to break the dormancy after storage to activate enzymes that stimulate germination. The use of inappropriate germination methods will reduce the success rate of coffee seed germination [18].

The previous limitations can be overcome by the progress and optimisation of biotechnological techniques in plant breeding, which enable the efficient propagation of many healthy plants in short periods of time. The efficiency of tissue culture regeneration protocols is influenced by several factors, including culture media composition, genotype and environmental conditions [19]. Specifically, light plays a crucial role in plant development [20]. 

The light spectrum is decisive in the germination and development of different plants such as arable weeds, herbaceous plants, Gesneriaceae family seeds, since light acts as a crucial stimulus due to the numerous photoreceptors present in these plants [21]. Light quality (spectral composition) is especially important as it provides energy for photosynthesis and influences signaling pathways that regulate plant development in the complex process of photomorphogenesis, which occurs during seed germination, seedling development, and the transition from vegetative to anthesis (photoperiodic phenomenon). The use of specific wavelengths within the visible spectrum has been reported with a significant impact on seed germination, mainly the positive effect of red light (660 nm), and also the effect of blue light (460 nm) on morphogenic processes such as de-etiolation [22].

In general, the light signaling pathway plays a crucial role in the growth, development, and adaptation of plants to their environment [22]. A number of unique photoreceptors with different wavelength absorption spectra and biochemical properties have been used to precisely delineate the light environment of plants [23]. These photoreceptors can be classified in to five classes according to the wavelength of light they absorb [24]. Numerous studies have shown that the use of red light has contributed significantly to plant germination and development. The characteristic of light, such as wavelength, direction, intensity and duration provide plants with signals that they monitor through highly sensitive photoreceptor and translate into cellular signals that affect endogenous growth and differentiation control mechanisms [25]. Red light is a component of visible spectrum that promotes plant growth due to the presence of photoreceptors that makes the photosynthetic process more efficient [26]. In habanero pepper plants, red light favored fruit yield and stomatal frequency [27]. Monochromatic red light favored the concentration of photosynthetic pigments, Mg and Mn an induce lower concentration of P and Cu. In some studies, it has been observed that the leaf area expansion of plants grown under red light was higher compared to other coloured cover, as well as in the uncovered control [28]. Red light promotes greater stem length, due to the action of phytochromes, which promotes cell division and extension, promoting stem elongation of plants [29,30].

Within the light spectrum, photosynthetically active light PAR (photosynthetically active radiation) stands out as the most influential, as it directly involved in plant development processes, stimulating flowering, stem elongation and both leaf and root development. In addition, the variation of wavelengths throughout the daily cycle and at different growth stage affects plant properties, including appearance, colour, aroma, taste, pharmaceutical and nutritional value [31].

Therefore, the objective of this research is evaluating the effect of light quality independently on the germination and development of coffee plants in order to optimise a propagation protocol.

## 2. Results

In this study, four- and nine-month-old *Coffee arabica* seeds were used and evaluated in two different stages. In the first stage, germination was evaluated and germination rate indexwas calculated in the first 30 days. In the second stage, seedling development parameters were evaluated.

**Figure 1:**
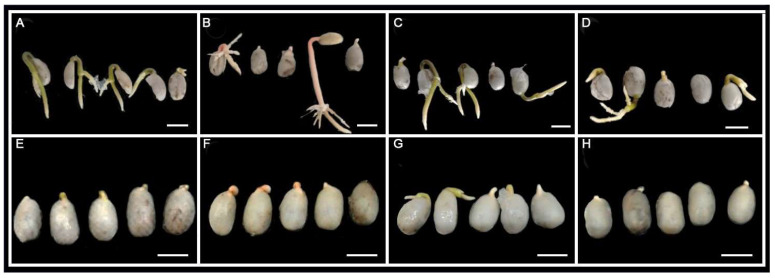
Germination effects of different wavelength at day 30. (**A**–**D**) 4-month experiment; (**E**–**H**) 9-month experiment; (**A**,**E**) Control, white light length; (**B**,**F**) Treatment 1, dark; (**C**,**G**) Treatment 2, red light length; (**D**,**H**) Treatment 3, blue light length. Bar 0.5 cm.

During the evaluation of coffee seed germination, the time at which the radicle breaks the seed testa was used as the end of germination criterion (Figure 1). In general terms, it was observed that coffee seeds germinated faster under white light photoperiod for 4-moth-old seeds, while for 9-month-old seeds the red-light photoperiod was more efficient. Regarding the age of the seeds, the 4-moth-old seeds showed a faster germination, presenting germination rate index (GRI) values between 5.20 and 20.05 times higher compared to the 9-moth-old seeds (Table 1, GRI coefficient). After sowing in the culture medium under in vitro condition, it could be observed that 4-moth-old seeds showed an onset of germination from the fourth day onwards. In contrast, the 9-moth-old seeds showed a later onset of germination, in the case of treatment 2 (red light) some seeds started to germinate on day 17, while in the other treatments some of the seeds started germinating on day 24. These results are reflected in the germination speed index values in Table 1, where higher values indicate that seed germination started earlier.

In relation to germination, it was observed that the 4-month-old seed show a germination percentage of 96.67% in the T0 treatment (white light), follow with by the T2 treatment (red light) with 93.33% germination. On the other hand, the blue light and dark treatment recorded the lowest germination percentage with 86.67% and 76.67%, respectively. In the case of 9-month-old seeds, the treatment with the highest germination percentage where T2 (red light) and T3 (blue light), with 65% and 62% germination, respectively (Table 1). It was observed that the germination percentage was 1.39 to 1.84 times higher in all treatments using 4-month-old seeds compare the treatment using 9-month-old seeds (Table 1, germinations coefficient). These results are also a reflection of the values observed in the germination speed (Table 1), science if the seeds germinate in the first days after being established in the culture medium, on the 30 th day after imbibition they will present a greater development, as can be seen in the Figure 1, the 4-month-old seeds show hypocotyl development and in the case of the root system some of the seed present secondary roots (Figure 1A–D). In the case of the 9-month-old seeds, can be observed that one day 30, many of the seeds have barely started germination (Figure 1E–G) and others have not yet germinated (Figure 1H).

In terms of seedling development stage, 4-month-old seeds under the T3 (blue light) treatment show it the highest growth parameter, followed by the T0 (white light) treatment and the T2 (red light) treatment. The T1 (dark) treatment showed the lowest values for the most parameters, except for the stem length, switch reached approximately 5.39 cm (Table 1). For the 9-month-old seeds, the control (T0) shows with the highest developmental parameters, except for stem length, which was approximately 3.65 cm (Table 1). After T0 (white light), the T2 (red light) treatment showed the highest values in development parameters.

During the propagation of micro cuttings one of the parameters of great interest in the production of nodes, during this experiment it was observed that this parameter was higher in the case of plants development in red light (T2) (Table 2). Therefore it is advisable to use red light to increase the production of number of nodes, in case of looking for propagation by cuttings.

**Figure 2:**
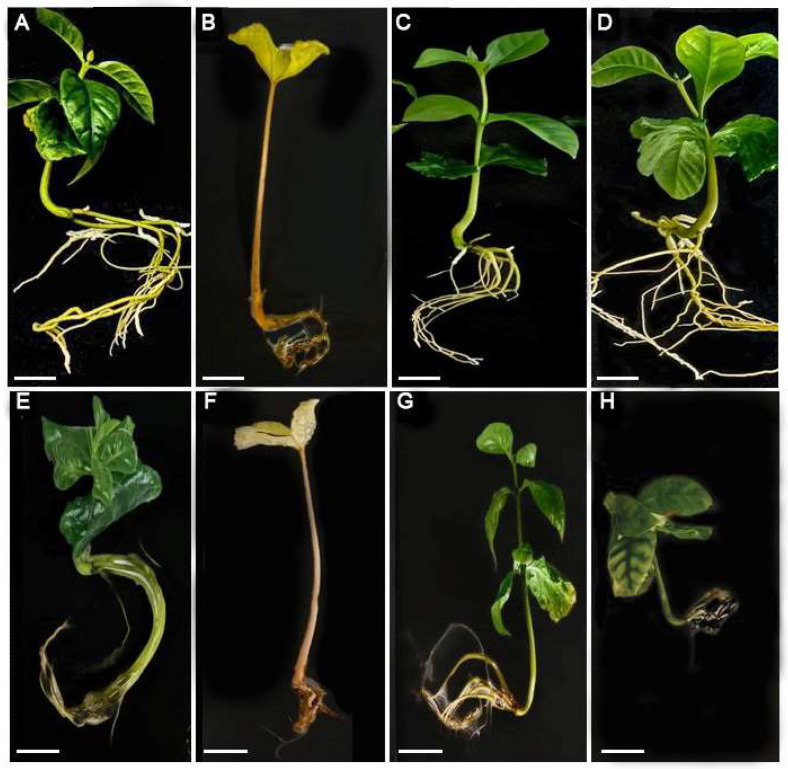
Effects of light quality of coffee sitting development under in vitro condition at 90 days of culture. (**A**–**D**) 4-month-old seeds; (**E**–**H**) 9-month-old seeds; (**A**,**E**) Control, White light; (**B**,**F**) Treatment 1, dark; (**C**,**G**) Treatment 2, red light; (**D**,**H**) Treatment 3, blue light. Bar 1 cm.

It is worth noting that the plans of treatment T0, T2 and T3 have a vigorous appearance with green leaves, comparison to treatment T1, where the seedling showed symptoms of etiolation. In general, seedling that continued their development in darkness proceed to skotomorphogenesis [32], showing elongated hypocotyls with a curvature at the stem apex and non-expanded cotyledons (Figure 2). In this case, the plastids of the etiolated plants have colourless plastids (without Chlorophyll and other photosynthetic pigments). In the absence of light, chlorophyll synthesis does not work for the transformation of its precursor, photo-chlorophyll into chlorophyll, is dependent on then enzyme PORA (protochlorophyllide oxidoreductase A) whose expression is regulated by light, therefore, PORA mRNA and protein are present at low level in the presence of light and present high value in dark condition [33,34].

## 3. Discussion

Although the sexual method has been the traditional option for coffee plant propagation, it has significant limitation due seeding damage and it’s slow germination rate in the field, a process that can be affected by climatic condition, as well as damage to seed integrity by microorganism and insect [35]. Therefore, in this study, the sGRI and percentage of germination of 4 and 9 month-old coffee seeds were evaluated under controlled condition in order to make the germination process more efficient of coffee seeds.

In this work, it was observed that 9 month old seeds decrease the germination percentage by 1.39 to 1.84 times compared to 4 month old seeds. This difference may be due to the fact that coffee seeds are considered recursive trends because they cannot be stored in dry condition for a long-term without losing their viability, they must be sown immediately after harvesting or kept in controlled humidity condition to conserve their germination capacity, which is why all processing, including pulping, sorting an experimental setup, must be short term because seeds lose water easily, initiating seed deterioration at very high rates [36], unlike orthodox (desiccation tolerant) seeds, seeds are acquiring longevity, which is the total time during which dry seeds remain viable [37].

This effect can be observed in the research work reported by Nasiro and collaborators [38] in which it was observed that coffee seeds with a moisture content of up to 12% start under ambient condition for six months were those that presented the lowest germination percentage with 43.67% in greenhouse condition, in which decrease in germination percentage was attributed to prolonged storage of the seeds, as well as temperature change, causing the ageing of the seeds which is reflected in a reduction of the radical emergency capacity [38,39,40]. Therefore, seed storage time exerts a significant influence and is considered to be a highly relevant factor inside viability. Coffee seed is capable of germinating inmediately after harvesting, so it should be used as soon as possible [41]. As a storage time elapses, its germination percentage decreases. Therefore, it is recommended to use freshly harvested seeds, to obtain a higher germination percentage, as the storage time elapses, the ability of the seeds is considerably reduced. Under field condition, germination loss during the first three months of storage is not significant; after three months, 70–75% germinates, after five months only 50% and after nine months 20–30% germination [16].

In coffee seed germination, beside of observing that the age of the seed is a factor that affect the germination process, there are other factors such as the quality of light. Light is a factor that regulate various plant processes, because the plants perceive light through various photoreceptors, which have the ability to detect different wavelength affecting the morphogenesis of the plant [42]. In this study it was observed that coffee seeds showed higher germination percentage in a red-light quality (T2) regardless of seed age. These are also observed in lettuce seeds, the application of red light promotes germination of the 100% [43]. In some research it is mentioned that the highest number of germinated seeds occurred under red light condition, preceded by red-light [44]. In *Arabidopsis* it has been confirmed that the seeds germination process is strongly influenced by phytochromes B (PhyB), which is the main photoreceptor responsible for germination in response to red light [45]. In both studies, it was found that red light showed the highest percentage, an outstanding speed in germination, which may suggest that in the case of coffee seeds could be involved phytochromes that promote germination in a red light quality.

In terms of plant development, the treatments T0, white light containing the visible spectrum (400 to 700 nm), as well as T2 and T3 under the wavelength of 700 nm (red light) and 400 nm (blue light) respectively, presented the highest values in most of the development parameter evaluated. This is due to the fact that the vast majority of plants absorb light more actively and efficiently in the red (600–700 nm) and blue (400–500 nm) regions, which correspond to the maximum light absorption peaks of photoreceptors (phytochromes, cryptochromes, phototropins and SEITLUPE proteins) and photosynthetic pigments that are part of the photosynthetic apparatus [27]. Red light was the most favourable for germination and the development of the seedlings in coffee. Numerous studies have shown that the use of red light has contributed significantly to plant germination and development [27,28]. Red light promotes greater stem length, due to the action of phytochromes, which promotes cell division and extension, promoting stem elongation of plants [29,30]. In the case of blue light, previous studies on wheat (*Triticum aestivum*) and barley (*Hordeum vulgare*) seeds have shown an inhibitory effect on seed germination [46,47]. In barley seeds, blue light induces the expression of the 9-cis-epoxycarotenoid dioxygenase gene and represses the expression of the ABA 8′-hydroxylase gene, which encode biosynthetic and catabolic enzymes respectively, which are key to regulating the endogenous ABA content [48]. In this study, it was observed that coffee seeds had a lower germination percentage under blue light (T3) compared to red light (T2), although there were no significant differences between both treatments. This response was also observed in *Brassica napus* seeds, where seeds exposed to red light have the highest percentage of germination, while seeds under blue light have a lower percentage of germination, evidencing a negative effect [48]. However, in dicotyledonous seeds such as Stevia, blue light promotes germination [49]. Generally, more studies are needed to elucidate the mechanisms and role of blue light in the germination of dicotyledonous seeds. In this study, although seed germination is lower under blue light compared to red light, it does not present an inhibitory effect compared to white light (T0), which in our case was used as a control.

## 4. Materials and Methods

Material vegetal. Coffee (*Coffea arabica* var. Typica) seeds were collected from backyard gardens located at the coordinates (20°24′44″ N 103°23’30″ W), Municipality Tlajomulco Zúñiga, Jalisco.

The seeds were washed with water, manually pulped (removed of pericarp, pulp, pectin layer and parchment) and subjected to a 10% chlorine solution with 1 mL L^−1^ tween 80 for 15 min, then rinsed with water and dried in a tray dryer (Polinox model TABIM094) at a temperature of 30 ± 2 °C for 24 h, then stored in glass bottle at room temperature (approximately 25 ± 2 °C) for later use.

Preparation of culture medium and seed establishment under in vitro conditions. For the establishment of the in vitro condition, 9-month and 4-month seeds age after harvesting were used, these were depulped and dried, the endocarp (parchment) was manually removed and subjected to a disinfection process under aseptic condition with a sterile solution, the seeds were placed in a disinfectant solution with benomyl (1 g L^−1^) and streptomycin (0.3 g L^−1^) for 20 min, then rinsed 3 times with distilled water, then place in a 30% solution of commercial chlorine (Cloralex) for 20 min and then rinsed 3 times with distilled water, then placed in 70% ethanol for 2 min and again rinsed 3 times with distilled water. FThis solution was then transferred to another solution of 0.1 g L^−1^ ascorbic acid and 0.15 g L-1 citric acid for 1 min and transferred to semisolid medium in dark condition at 27 ± 2 °C.

Semisolid ZG culture medium was prepared for coffee seed germination [50], supplemented with 0.1 mg L^−1^ of naphthalenacetic acid (NAA) and 0.5 mg L^−1^ of kinetin, 30 g L^−1^ of sucrose and 4 g L^−1^ of gelrite were also added. The pH was adjusted to 5.8 before addition of the gelling agent, then sterilised at 120 °C at 117 kPa for 20 min.

Light quality experiment. Two experiments were carried out using seeds of different ages. In the first experiment, seeds that were 9 months old after harvesting were used, while in the second experiment, seeds that were 4 months old were used. The seeds were disinfected and placed in MS culture medium and subjected to four different light qualities: T0 (Control): white light, White LEDs with a broad wavelength of 400–700 nm; T1: Dark; T2: Red light, Red LEDs with a wavelength of 660 nm; T3: Blue light, Blue LEDs with a wavelength of 460 nm. Irradiation intensity of artificial light was set to 40 μmol m^−2^ s ^−1^. All treatments were maintained in the following condition 25 ± 2 °C, photoperiod 16 h light during the experiment.

The evaluation was divided into two stages, germination and seedling development. Germination was evaluated every third day until the 30 th day, when the seed germinated when the radicle broke the testa. As for the evaluation of the seedling, the following parameter of seedling development was evaluated in the third month after seed germination: including number of nodes (NN), number of true leaves (NH), number of roots (NR), length of main root (LR) and length of stem (LS). It should be clarified that the LS parameter was considered as the length from the base of the stem to the apex of the seedling.

Germination rate index (GRI). The number of germinated seed was counted every third day. Root protusion was considered as a germination criterion. The GRI was calculated according to Maguire [51].

$$GRI=\sum \frac{n_{i}}{t}$$

*GRI* = Germination rate index
*n_i_*= Number of seeds germinated on day
*t*= Germination time from sowing to germination of the last seed

Experiment analysis. Seed germination and development of coffee seedlings were evaluated. The experimental design was completely randomised blocks, the data presented correspond to the mean of eight replicates, each with 5 seeds. The data was subjected to an analysis of variance (ANOVA). Comparison of mean was determined by the LSD-Fischer test (*p* < 0.05). 

## 5. Conclusions

Seeds with a lower storage age (4 month) showed a higher germination rate and germination percentage compared to 9-month-old seeds. In general terms, the 4-month-old seed in the white light (T0) and red light (T2) showed a higher germination rate, while for the 9-moth-old seeds, the red light (T2) and de blue light (T3) treatments showed the highest values for germination rate and germination percentage. 

In the case of treatment T1, it was observed that the plants showed an etiolated phenotype, with an excessive elongation of the stem extending to the surface of the container, which resulted in the fracture of the plant and its subsequent death. As for blue light (T3), a germination percentage of 57.50% was recorded, which positioned it as the most outstanding in this aspect. In turn, the T2 treatment showed intermediate results in the parameters evaluated during the development stage compared the other treatments. 

Red light was the most favourable for germination, reaching a percentage of 67.5%. When continuing with the experiment and evaluating the development of the seedlings, it was observed that this same treatment exhibited greater growth in several development parameters: the number of leaves (NH), the number of nodes (NN) and the length of the stem (LS). In this research, it was described that in coffee (*Coffea arabica*) red light had a beneficial effect on seed germination and seedling development, greater than conventional white light. And also that blue light did not have an inhibitory effect on germination as described for other crops.

## Figures and Tables

**Table 1:** Evaluation of GRI and germination of coffee seeds after 30 days of in vitro culture.

T	GRI ^1^			%G ^1^		
	4 Months+	9 Months+	GRI_4_/GRI_9_	4 Months+	9 Months+	%G_4_/%G_9_
T0	4.41 ± 0.27 ^a^	0.22 ± 0.02 ^bc^	20.05	96.67 ± 3.33 ^a^	52.50 ± 2.50 ^b^	1.84
T1	3.39 ± 0.38 ^ab^	0.22 ± 0.01 ^bc^	15.41	76.67 ± 3.33 ^b^	52.50 ± 2.50 ^b^	1.46
T2	3.64 ± 0.35 ^ab^	0.70 ± 0.07 ^a^	5.20	93.33 ± 3.33 ^a^	65.00 ± 2.89 ^a^	1.44
T3	3.15 ± 0.40 ^b^	0.36 ± 0.03 ^b^	8.75	86.67 ± 3.33 ^ab^	62.50 ± 2.50 ^ab^	1.39

^1^ GRI -Germination rate index, %G-Germination percentage. T. Treatment, T0 (White light), T1 (Dark), T2 (Red Light), T3 (Blue Light), %G4/%G9 (Germination Quotient). +The table shows the means with their standard error and the different letters between columns indicate significant differences with the LSD test (*p* < 0.05).

**Table 2:** Developmental parameters of coffee seedlings exposed to different light qualities at 90 days of cultivation.

	**Seeds 4-Months-Old**				
	**NH ^1,2^**	**NR ^1,2^**	**RL ^1,2^**	**NN ^1,2^**	**SL ^1,2^**
C	2.03 ± 0.38 ab	2.63 ± 0.19 ab	6.98 ± 0.86 a	2.06 ± 0.40 a	5.74 ± 0.50 ab
T1	0.00 ± 0.00 c	2.54 ± 0.14 b	4.42 ± 0.47 b	0.00 ± 0.00 b	5.39 ± 0.40 ab
T2	1.68 ± 0.22 b	2.25 ± 0.17 b	5.61 ± 1.01 ab	2.89 ± 0.46 a	4.69 ± 0.36 b
T3	2.65 ± 0.36 a	3.33 ± 0.51 a	7.37 ± 0.81 a	2.20 ± 0.41 a	6.24 ± 0.70 a
	**Seeds 9-months-old**				
C	2.09 ± 0.05 a	2.31 ± 0.20 ab	4.28 ± 0.47 a	2.09 ± 0.50 a	3.65 ± 0.38 b
T1	0.00 ± 0.00 b	1.93 ± 0.22 b	1.53 ± 0.15 c	0.00 ± 0.00 b	3.25 ± 0.42 b
T2	2.36 ± 0.47 a	2.79 ± 0.23 a	2.93 ± 0.61 b	2.71 ± 0.53 a	6.04 ± 0.52 a
T3	0.62 ± 0.22 b	2.07 ± 0.19 b	2.16 ± 0.21 bc	0.62 ± 0.22 b	2.99 ± 0.26 b

^1^ NH (number of leaves), NR (numbers of roots), RL (root length), NN (number of nodes), SL (steam length). ^2^ Table shows the means with their standard deviation, the different letters between columns indicate significant difference with the LSD test (*p* < 0.05).

## Data Availability

Data are available upon request.
